# Supplementary material for: Incidence, Risk Factors and Impact on Clinical Outcomes of Bloodstream Infections in Patients Hospitalised with COVID-19: A Prospective Cohort Study
Source: Antibiotics (Basel). 2021 Aug 24;10(9):1031. doi: 10.3390/antibiotics10091031 (PMC8470708; doi:10.3390/antibiotics10091031)
Supplement: Supplementary file 1 [file antibiotics-10-01031-s001.zip › antibiotics-1286568-supplementary.pdf]

SUPPLEMENTARY TABLE S1. List of pathogens isolated from blood cultures considered contaminants and excluded from the study

Pathogens	N=92
Staphylococcus epidermidis	38
Staphylococcus hominis	28
Staphylococcus haemoliticus	9
Staphylococcus capitis	6
Staphylococcus pettenkoferi	3
Staphylococcus warneri	2
Bacillus clausii	1
Bacillus spp	1
Bulkholderia cepacia	1
Corynebacterium striatum	1
Gemella Haemolysans	1
Streptococcus mitis/oralis	1

SUPPLEMENTARY TABLE S2. List of other microbiological findings in patients included in the study

Other microbiological findings, N (%)	Patients without BSI N=1,282 (94.9%)	Patients with CA/HCA-BSI N= 18 (1.3%)	Patients with HA-BSI N= 51 (3.8%)	*p* value	Overall N=1,351
Respiratory samples (sputum/BAS/BAL)	51 (3.9%)	0 (0.0%)	10 (19.6%)	<0,001	61 (4.5%)
Urine cultures	110 (8.6%)	6 (33.3%)	23 (45.1%)	<0,001	139 (10.3%)
Positive Pneumococcal urinary antigen[a]	44 (3.4%)	2 (11.1%)	1 (1.9%)	0,111	47 (3.5%)
Positive Legionella urinary antigen[b]	4 (0.3%)	0 (0.0%)	0 (0.0%)	0,929	4 (0.3%)
Positive for Influenza[c]	0 (0.0%)	0 (0.0%)	0 (0.0%)		0 (0.0%)
Serology for atypical pulmonary pathogens[d]					
Chlamydia	2 (0.2%)	0 (0.0%)	0 (0.0%)	0,934	2 (0.1%)
Mycoplasma	34 (2.7%)	1 (5.6%)	1 (1.9%)	0,670	36 (2.7%)
MDR colonization	15 (1.2%)	2 (11.1%)	1 (2.0%)	0,001	18 (1.33%)
Blood cultures positive for contaminants	65 (5.1%)	3 (16.7%)	14 (27.5%)	<0,001	82 (6.1%)
Other	11 (0.9%)	3 (16.7%)	6 (11.8%)	<0,001	20 (1.5%)

SUPPLEMENTARY TABLE S3. Uni- and multi-variable analysis on factors associated with in-hospital mortality

	OR	95%CI		*p* Value	AOR*	95%CI		*p* Value
**Age**, per 10 years older	1.98	1.78	2.19	<0.001	2.09	1.83	2.37	<0.001
**Gender, male** (vs. female)	1.26	0.97	1.63	0.082	1.59	1.16	2.18	0.004
**Charlson age unadjusted**, per one-point raise index	1.24	1.17	1.31	<0.001	1.14	1.08	1.21	<0.001
**CRP >60 mg/L** (vs <=60 mg/L)	3.65	2.79	4.79	<0.001	2.58	1.856	3.57	<0.001

**D-Dimer > 1.000 ng/mL** (vs. <=1.000 ng/mL)	3.03	2.18	4.22	<0.001	1.62	1.11	2.39	0.014
**COVID-19 severity at admission** (vs mild/moderate)								
Severe	2.86	2.14	3.83	<0.001	2.35	1.70	3.23	<0.001
Critical	14.26	6.85	29.68	<0.001	14.11	5.90	33.77	<0.001
**Anti-inflammatory treatment** (steroids and/or immunomodulators)	1.00	0.78	1.28	0.983	1.15	0.81	1.63	0.434
**Calendar Period of Admission, Aug-Nov 2020** (vs Feb-Jul 2020)	0.57	0.45	0.73	<0.001	0.47	0.33	0.65	<0.001
**CA/HCA-BSI**	1.51	0.56	4.05	0.414	1.27	0.411	3.90	0.681
**HA-BSI**	1.38	0.75	2.53	0.297	1.29	0.65	2.54	0.463

SUPPLEMENTARY TABLE S4. STROBE Statement

STROBE Statement—Checklist of items that should be included in reports of ***cohort studies***

	Item No	Recommendation	Page No
**Title and abstract**	1	(*a*) Indicate the study's design with a commonly used term in the title or the abstract	1,2
		(*b*) Provide in the abstract an informative and balanced summary of what was done and what was found	2
**Introduction**			
Background/rationale	2	Explain the scientific background and rationale for the investigation being reported	3
Objectives	3	State specific objectives, including any prespecified hypotheses	3
**Methods**			
Study design	4	Present key elements of study design early in the paper	3
Setting	5	Describe the setting, locations, and relevant dates, including periods of recruitment, exposure, follow-up, and data collection	3, 4
Participants	6	(*a*) Give the eligibility criteria, and the sources and methods of selection of participants. Describe methods of follow-up	3, 4
		(*b*) For matched studies, give matching criteria and number of exposed and unexposed	-
Variables	7	Clearly define all outcomes, exposures, predictors, potential confounders, and effect modifiers. Give diagnostic criteria, if applicable	3, 4
Data sources/ measurement	8*	For each variable of interest, give sources of data and details of methods of assessment (measurement). Describe comparability of assessment methods if there is more than one group	4, 5
Bias	9	Describe any efforts to address potential sources of bias	5, 6

Study size	10	Explain how the study size was arrived at	NA
Quantitative variables	11	Explain how quantitative variables were handled in the analyses. If applicable, describe which groupings were chosen and why	5, 6
Statistical methods	12	(*a*) Describe all statistical methods, including those used to control for confounding	5, 6
		(*b*) Describe any methods used to examine subgroups and interactions	NA
		(*c*) Explain how missing data were addressed	NA
		(*d*) If applicable, explain how loss to follow-up was addressed	-
		(*e*) Describe any sensitivity analyses	-
**Results**			
Participants	13*	(a) Report numbers of individuals at each stage of study—eg numbers potentially eligible, examined for eligibility, confirmed eligible, included in the study, completing follow-up, and analysed	6
		(b) Give reasons for non-participation at each stage	6
		(c) Consider use of a flow diagram	-
Descriptive data	14*	(a) Give characteristics of study participants (eg demographic, clinical, social) and information on exposures and potential confounders	Table 1
		(b) Indicate number of participants with missing data for each variable of interest	-
		(c) Summarise follow-up time (eg, average and total amount)	8, 9
Outcome data	15*	Report numbers of outcome events or summary measures over time	8, 9<br>Table 2
Main results	16	(*a*) Give unadjusted estimates and, if applicable, confounder-adjusted estimates and their precision (eg, 95% confidence interval). Make clear which confounders were adjusted for and why they were included	8, 9<br>Table 4
		(*b*) Report category boundaries when continuous variables were categorized	Table 1, 2
		(*c*) If relevant, consider translating estimates of relative risk into absolute risk for a meaningful time period	-
Other analyses	17	Report other analyses done—eg analyses of subgroups and interactions, and sensitivity analyses	-
**Discussion**			
Key results	18	Summarise key results with reference to study objectives	9
Limitations	19	Discuss limitations of the study, taking into account sources of potential bias or imprecision. Discuss both direction and magnitude of any potential bias	11,12
Interpretation	20	Give a cautious overall interpretation of results considering objectives, limitations, multiplicity of analyses, results from similar studies, and other relevant evidence	9-12
Generalisability	21	Discuss the generalisability (external validity) of the study results	11,12
**Other information**			

Funding	22	Give the source of funding and the role of the funders for the present study and, if applicable, for the original study on which the present article is based	13
